# Paraquat at 63—the story of a controversial herbicide and its regulations: It is time to put people and public health first when regulating paraquat

**DOI:** 10.1186/s12889-025-23830-w

**Published:** 2025-09-24

**Authors:** Leah Utyasheva, Prabath Amarasinghe, Michael Eddleston

**Affiliations:** 1 Centre for Pesticide Suicide Prevention, University of Edinburgh, Edinburgh, UK; 2 Pharmacology, Toxicology & Therapeutics, University/BHF Centre for Cardiovascular Science University of Edinburgh, Edinburgh, UK

**Keywords:** Paraquat regulation, Paraquat poisoning, Paraquat ban, Paraquat mortality, Restrictions on use

## Abstract

**Background:**

Paraquat is one of the most widely used herbicides in the world, despite its high human toxicity and the overwhelming evidence of associated high morbidity and mortality. Due to the significant public health implications of the use of paraquat, there have been calls to severely restrict or ban it in many countries. In this paper, we aim to investigate the regulatory status of paraquat at the national, regional, and international levels, discuss the successes and challenges of regulatory implementation, and review the impact of regulation on the incidence of poisoning and death.

**Methods:**

We conducted a systematic review of articles on the regulation of the herbicide paraquat. The review concentrated on interventions to mitigate the negative public health impact of paraquat use. To complement our findings, we also conducted region-wise and country-wise searches on paraquat regulations. We collected information on paraquat regulations and restrictions and regulatory aspects of regulation implementation.

**Results:**

At least 74 countries do not authorise paraquat in their markets, with bans, phase-outs, and withdrawals from the market. National and regional bans and phase-outs were effective at reducing paraquat poisoning and deaths. Restrictions on the use and application of paraquat, however, did not always result in a significant reduction in poisoning or suicide mortality, and many countries introduced bans after restrictions proved ineffective.

**Conclusion:**

Our review highlights several important elements for the success of the implementation of these regulations, with reductions in harm and no effect on agriculture. It is now time for national, regional, and international authorities to pay attention to the scientific evidence of human toxicity and put people’s lives and health ahead of economic and business considerations in regulating paraquat. Its use should be replaced by other of weed control. This should be a public health priority.

**Supplementary Information:**

The online version contains supplementary material available at 10.1186/s12889-025-23830-w.

## Introduction

Since the introduction of synthetic chemical pesticides after World War II, global pesticide production and use have increased manifold. Unfortunately, this has been accompanied by multiple public health problems, including an estimated 14 million premature deaths, extensive food and environmental contamination, chronic diseases from low-level exposure, and severe losses of biodiversity and insects affecting pollination and food production [1–3].

Much of the recent increase in use, with accompanying public health issues, is due to the growing demand for herbicides, particularly in low- and middle-income countries (LMICs). From 2000 to 2017, global herbicide exports nearly quadrupled in volume, increasing 30% more than exports of all other pesticide classes [4]. In 2019, herbicides made up 43% of the global pesticide market by value, exceeding insecticides (27%) and fungicides (27%) [5]. This growth is driven by factors such as population growth and a reduction in farmland area, rising rural outmigration, decreasing availability and increasing labor costs for hand weeding, and demands for urban and public weed control, leading to a situation where herbicides replaced labor in many agricultural and urban environments [6]. This trend is supported by the shift in pesticide production from the global North to the global South and increasing South-South import flows, with many herbicides produced off-patent in Asia [7].

Paraquat dichloride is the active ingredient of one of the oldest and most widely used herbicides in the world. It was first synthesised in 1882 when it was known as methyl viologen and used as a chemical dye [8]. Its herbicidal properties were discovered in 1955; it started to be produced industrially in the UK in 1961 by Imperial Chemical Industries (ICI) and became commercially available in 1962 under the commercial brand Gramoxone. After ICI’s restructuring in 1993, paraquat was produced by the newly formed Zeneca [9]. In 2000, Zeneca was sold to the Swiss company Novartis Agrochemicals, which changed its name to Syngenta AG, with headquarters in Switzerland. In 2016, Syngenta was purchased by ChemChina, a Chinese state-owned company; in 2021, it sold paraquat in 28 countries, accounting for a quarter of global sales of paraquat [10]. Syngenta, however, is not paraquat’s only manufacturer; presently, there are 377 companies globally holding valid registrations for the production of generic paraquat [11].

Paraquat acts as a nonselective contact herbicide, destroying the green parts of plants without affecting the roots. It is popular among farmers because of its wide range of herbicidal activities and lack of residual effects as a result of rapid inactivation on contact with soil [8]. However, despite being classified as class II (moderately toxic) by the World Health Organisation (WHO) [12], based on oral toxicity data in rats, it is highly toxic to humans under all modes of exposure, with particularly low lethal oral dose. There is no antidote.

The first reports of human poisoning appeared in 1964, as early as two years after the launch of the product [13]. Since then, paraquat has been responsible for hundreds of thousands of intentional and accidental poisonings worldwide, many of which are fatal [14–17]. Paraquat damages the lungs, kidneys, liver, cardiac system, and other bodily systems [18–20]. Its long-term links with Parkinson’s disease are vigorously debated, with the industry disputing these links. More than 400 lawsuits against Syngenta are pending in the U.S. and Canada filed by individuals diagnosed with Parkinson’s disease [21]. The acute poisonings associated with paraquat are not disputed– they are widely known and have led to a series of measures that are the main topic of this paper.

Lives lost and health ruined by paraquat have led to calls for its ban at the national, regional, and international levels. Restrictions and bans on its use in major agriculture-producing countries—China, Brazil, and Thailand—decreased the overall use and sales of the herbicide in the past several years [11]. Such withdrawals have not affected agricultural output [22–24]. Paraquat is not authorised in European countries; its use is strictly restricted in high-income countries (HICs), where it is still permitted. The largest producers of paraquat, the United Kingdom and China, banned its domestic use but continue to export it to other countries.^1^

At 63 years of age, the use of paraquat is still controversial– it is one of the most heavily researched and regulated pesticides and has attracted the attention of the global community and policymakers.^2^ Despite losing its status as the world’s second most popular herbicide, paraquat is still widely used. With the increase in the global price of glyphosate and glufosinate (the most popular alternatives to paraquat), and the continued controversy over glyphosate’s carcinogenicity and crops’ spreading resistance to it, paraquat’s fortunes may yet change [6, 11].

The purpose of this paper is to analyse the status of the herbicide paraquat in national, regional, and international regulatory contexts and to discuss the types of regulations governing its use and distribution and the evidence of its impact on public health. We posit that, with the overwhelming evidence of paraquat’s toxicity and widespread harm to health, it is time that national, regional, and international authorities pay attention to the scientific evidence of human toxicity and put people’s lives and health ahead of economic and business considerations in regulating paraquat. Its use should be replaced by other methods of weed control [25]. This should be a public health priority.

## Methods

**Fig. 1:**
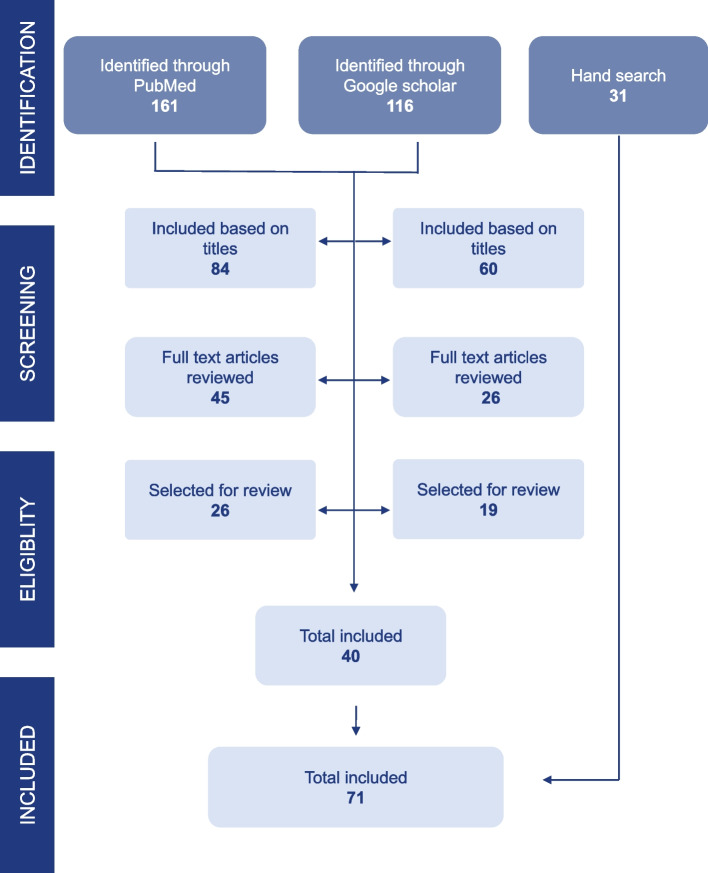
The PRISMA Flow diagram for the study

We conducted a systematic review of articles on the regulations of the herbicide paraquat following the Preferred Reporting Items for Systematic reviews and Meta-Analysis (PRISMA) Guidelines [26]. The review concentrated on interventions to mitigate the negative public health impact of paraquat use. We searched PubMed and Google Scholar for English-language peer-reviewed articles published up to December 2022. For both, we conducted a title and abstract search. We searched the reference lists of eligible papers and conducted citation searches in Google Scholar to identify additional articles. We also identified research papers from our collections and checked relevant papers for additional studies (Figure 1). We included grey literature (thesis, national, regional, and international reports on paraquat regulations and pesticide regulations in general) as supplementary information on paraquat regulations. We searched for information on paraquat regulations on the website of the Rotterdam Convention on the Prior Informed Consent Procedure for Certain Hazardous Chemicals and Pesticides in International Trade (RC). The RC Secretariat receives notifications on pesticide restrictions and bans from member countries and has been discussing paraquat regulations since 2011.

The search terms used were ‘paraquat ban’, ‘paraquat regulation’, and ‘paraquat restriction’. To account for the fact that paraquat regulations could have been covered in papers describing general pesticide regulations, we also searched the terms “pesticide regulation”, “pesticide ban” and “pesticide restriction”. Inclusion of health or other response (health outcomes, poisoning rates, mortality rates, agricultural output, response of policymakers or response of stakeholders) were not considered important for this review. We excluded literature on the medical aspects of paraquat poisoning and case reports. We also excluded studies for which full texts were unavailable and papers in languages other than English. Two researchers independently screened the titles and abstracts and extracted the data, including locations, findings, and lessons learned. Table 1 shows the headings used for data extraction for the literature review.

**Table 1:** Headings for the systematic review literature

Authors	Date	Title	Journal/Publisher	Vol/Part	Pages	Countries covered	Reasons for regulations	Implementation	Lessons Learned	Recommendations	Comments	Notes

In addition to the literature review, we performed region-wise and country-wise searches on paraquat regulations to complement our findings. We concentrated on countries that have been identified as having regulations on paraquat. We collected information on paraquat regulations and restrictions and the regulatory aspects of regulation implementation. We searched the official websites of agencies responsible for pesticide registration from March 2022 to September 2023. Information was also obtained from the FAO’s legal database faolex.org. Where available, we checked national lists of banned and restricted pesticides to identify the regulatory status of paraquat. Table 2 shows information about countries that regulate paraquat and the types of regulations that exist.

**Table 2:** List of paraquat regulations

No	Countries	Date of regulations	Type of regulation/Issuing body	Reasons	Comments	References

There were no date limitations to the included sources. The literature search was performed in June-December 2022, with a final search done in December 2022.

The results are analysed according to geographic location and presented as national, regional and international case studies. For the purposes of this paper, we define “regional context” as relating to a particular area with similar social, cultural, climatic, and agricultural contexts within certain geopolitical boundaries. “International” is defined as pertaining to an area with global social, cultural, climatic, and agricultural contexts. National case study refers to a situation within a country or a state/province within a country.

## Results

### Documents identified

Following the PRISMA guidelines, we retrieved 277 articles from PubMed and Google Scholar and 62 documents from the grey literature. Of the 161 articles identified through PubMed, 77 articles were excluded based on the titles. Of the 84 articles included, 39 were then excluded after the abstracts were reviewed, and 45 articles were retrieved as full texts (two could not be obtained). Seventeen articles were excluded because they did not meet the criteria, leaving a total of 26 articles selected for the review. A total of 116 articles were identified through Google Scholar, and 56 articles were excluded based on the titles. Of the 60 articles included, 34 were excluded based on the abstracts, three were excluded based on the full-text articles, and four full texts could not be obtained, leaving nineteen articles for review. Overall, 40 articles were selected after excluding five duplicate articles. A further 31 documents remained from grey literature and personal collections. Therefore, the total number of selected articles for the review was 71 (Fig. 1).

Many of the papers were country-specific studies of paraquat regulations and their effect; others were general studies of pesticide regulations and their effects and implementation, which included discussions of paraquat regulations. Among the country-specific papers, the majority were case studies from Sri Lanka [27–31], South Korea [32–36], Taiwan [37, 38], Japan [24, 39], and Brazil [40, 41]. Several papers presented regional and global overviews [1, 23, 42–49].

The documents indicated that most countries that authorise paraquat have restricted its use, and at least 74 countries have removed it from domestic use (Supplementary file 1).

### Forms of regulation

The easy availability of paraquat was associated with high numbers of poisonings and deaths [17, 50–53]. Overall, the reviewed papers indicated high mortality of paraquat poisoning as the reason for paraquat’s regulations.

Three types of regulations were introduced by governments and sometimes industry to decrease poisonings and deaths. First, formulation changes were developed in the hope of making paraquat less toxic to humans. Second, restrictions on use, such as requiring prescriptions for sales, signature on purchase, and application by professional applicators were introduced. Third, bans and phase-outs– a period during which all products must be used and after which no more sales or use can happen– were implemented. Some countries used just one of these approaches; other countries used more than one approach, moving from one to another and often ending up banning the product because restrictions were not effective in reducing deaths.

Most of the reviewed literature reported the positive impact of paraquat regulations on the reduction of poisonings and deaths [35, 36]. The most impactful decreases in poisonings and suicide mortality were associated with bans and phase-outs of the herbicide [23, 54]. In South Korea, where paraquat was the most important agent for self-poisoning deaths and where the ingestion of pesticides accounted for one-fifth of suicides in 2006–2010, pesticide suicide mortality halved from 5.26 to 2.67 per 100 000 people after the ban on paraquat. It was estimated that, in 2013, the regulations were followed by 847 fewer pesticide suicides, a 37% reduction in rates [35, 36]. In Sri Lanka, a cumulative effect of pesticide bans, including a paraquat ban, was estimated to have prevented 93,000 suicide deaths in 20 years up to 2015 [29]. In Taiwan, the 2018 ban on the import and production of paraquat was associated with a 37% decrease in the pesticide suicide rate in 2019, with 190 fewer suicides [38].

Papers that discussed the economic impact of the regulations on paraquat did not report changes in crop productivity or economic losses associated with pesticide regulation [24, 38, 55].

### National stories

The literature review revealed that paraquat regulations frequently developed in the stages described before [a) formulation changes; b) restrictions on, e.g., use and sale; and c) bans and phase-outs].

#### Making paraquat ‘safer’

In the 1970 s, when evidence of poisoning began to mount, paraquat formulations were first changed with the addition of three'safening'agents to reduce the incidence and severity of poisoning, including additives to increase the survivability of ingestion (emetic and gelling agents), and deterrents to ingestion (stenching agent and dye). For example, ICI and its American partner Chevron Chemical Co. added color (blue dye to distinguish it from brown fizzy drinks and reduce unintentional poisoning), a stenching agent (a strong and deterring odour), and an emetic agent (to induce vomiting) to its paraquat formulation, measures that failed to reduce the lethality of poisoning [15, 56].

In Sri Lanka, a new formulation called Gramoxone Inteon, which was designed to reduce paraquat absorption and prevent severe poisoning was introduced by Syngenta in 2004 [57]. It contained a gelling agent to thicken the formulation, an increase in the amount of emetic to induce vomiting, and a purgative to speed its exit from the small intestine [27]. However, it failed to have a clinically significant effect on the case fatality of paraquat self-poisoning, only reducing the case fatality from 72.9% to 63.3% [27]. In 2008, the introduction in Sri Lanka of a reduced concentration paraquat product (from 20.0% to 6.5%) was associated with a greater reduction in mortality—it fell from 50 to 23% [27] but was still high compared to the case fatality rate of many other pesticides [55].

Before the eventual ban of paraquat in Taiwan in 2020, similar changes to paraquat’s formulation were introduced, including the addition of stenching agents, dyes, and emetics to deter people from ingesting it. However, this approach also failed to significantly reduce the number of fatal poisonings [37].

In Japan, an SL20 formulation of paraquat was registered in 1962. In the 1980 s, the high case fatality rate following self-poisoning with paraquat became a public health concern. In response, the government mandated a marked reduction in the concentration of paraquat solution, from 20 to 4.3% [29]. The dilution of paraquat products was modestly effective at reducing deaths [24], similar to what has been observed in Sri Lanka. However, in both Sri Lanka and Japan, the case fatality rate following paraquat poisoning has remained high compared to that following the use of other pesticides.

Lethal paraquat poisoning was a major issue in Samoa in the 1970s. An attempt to ensure the storage of pesticides in locked storage boxes was undertaken to showcase the approach of ‘safe storage’ as effective at preventing poisonings. However, reanalysis has indicated that it was the withdrawal of paraquat from the market due to the cost of imports that was associated with a marked decrease in deaths [52]. A large RCT in Sri Lanka subsequently demonstrated that encouraging household storage of pesticides is unlikely to be an effective approach [58].

#### Restrictions

Many countries report having restrictions on paraquat use. In the United States**,** where paraquat has been widely used since its first registration in 1964, paraquat products are classified as “restricted use pesticides”. This means that paraquat products can be purchased and used only by trained certified applicators [59] Table 3. The US EPA is currently reviewing paraquat. Its latest interim decision (2021) included the following restrictions:

**Table 3:** Restrictions on paraquat use in the US (2021)

• Limit aerial applications to a maximum of 350 acres per applicator per 24-h period for all uses except cotton desiccation
• Require a residential area drift buffer for all aerial applications
• Prohibit the use of human flaggers
• Prohibit pressurized handgun and backpack sprayer application methods
• Limit the maximum application rate for alfalfa to one pound of paraquat cation per acre
• Require enclosed cabs if the area treated in 24 h is more than 80 acres
• Require enclosed cabs or PF10 respirators if the area treated in 24 h is 80 acres or less
• Require a 7-day restricted entry interval (REI) for cotton desiccation
• Require a 48-h REI for all crops and uses except cotton desiccation
• Require mandatory spray drift management label language [59]

In Canada, before Syngenta voluntarily withdrew paraquat from the market in 2023, paraquat could be sold only in a closed-system package, used with a closed-transfer system for mixing and loading, and applied by groundboom [60]. In New Zealand, paraquat must contain effective emetics and stenching agents. There are regulations for buffer zones, spray droplet size, and single and annual application rates. Paraquat can be used only by certified handlers [61].

In Australia, paraquat is registered as a Schedule 7 poison according to the national registration standard. Its purchase, possession, and use require special authorization. It cannot be used for domestic or domestic garden purposes. The product formulation must be colored blue or green and contain a stenching agent [62].

In Japan in 1986, in addition to banning the SL20 formulation of paraquat and replacing it with a lower concentration combination product, the government introduced requirements for buyers to show their identification for purchase and sign their name when buying any paraquat products [24]. As a result of all these regulations and due to the reduced use of highly hazardous organophosphorus insecticides, the number of deaths from pesticide poisoning decreased from 2648 in 1986 to 221 in 2019, a 92% reduction over 33 years.

In the Dominican Republic, paraquat belongs to the “restricted use” category. The product must contain a sharp smell and distinctive color, a vomiting agent, and no more than 20% paraquat ion. It requires government authorization for product marketing, handling, and storage and must be sold in containers with volumes between one and 20 L [63].

In Uruguay, the Ministry of Livestock, Agriculture, and Fisheries prohibits the use of paraquat as a crop desiccant, requires a prescription, and limits the active ingredient concentration (not higher than 28% p/v), packaging size (between 1 and 30 L), and formulation color (blue) [64]. In Colombia, the General Directorate of Agricultural Services restricts paraquat to application by professional applicators and prescription sales; it can be used only for certain crops (potato, sugarcane, and forage legume seedbeds). Aerial application of paraquat is prohibited in Colombia [65].

Restrictions on the use and sale of the herbicide may require significant resources for implementation and prove to be more effective in countries with high regulatory and enforcement capacity. Many papers from LMICs mentioned difficulties in implementing such restrictions [30, 37, 51, 66–69]. For example, in India, while the Central Insecticide Board and Registration Committee approved paraquat for use on nine crops, it is reportedly being used for 25 crops, with multiple other violations of label requirements, leading to poisonings and deaths [70].

In countries with high numbers of paraquat poisonings and deaths, restrictions on the use and sale of paraquat appeared to have an insufficient effect on minimizing the negative impact on public health compared to bans and eventually leading to bans.

In 1999, the South Korean government restricted paraquat use by increasing the required qualifications for sellers and recording personal information from buyers; a new paraquat formulation to reduce paraquat absorption was also introduced [71]. Unfortunately, despite the implementation of restrictions on its use, the number of suicides involving paraquat did not decrease until after the ban in 2011–2012 [35, 36, 72]. Similarly, in Taiwan, restrictions on the use of paraquat did not significantly reduce suicide numbers before its eventual ban in 2020 (discussed below).

Ireland introduced restrictions on the sale and use of paraquat in the 1960 s and 1970 s to address the rising death rates from paraquat poisoning [23, 53]. Use was permitted by licensed dealers and agriculture-related occupations only, the number of retail outlets was reduced, farmers were educated, and safety labeling was enforced [23]. However, these restrictions were ineffective at reducing the number of paraquat suicides, and paraquat was later banned throughout European Union countries in 2007.

#### Phase-outs and bans

European countries were the first to ban paraquat due to its high acute toxicity, irreversible toxic effects, and risk of unintentional poisoning (Norway (1981), Sweden (1983), Hungary (1991), Austria (1993), Denmark (1995), and Finland^3^ (1996)) [73]. A regional-level European Union (EU) wide ban on paraquat applicable to all 27 member countries in 2007 

In Brazil, in 2015, the National Health Surveillance Agency (ANVISA) concluded that paraquat should be banned due to the high incidence of acute poisoning and the association with Parkinson's disease [41, 74]. In 2017, the Collegiate Directorship Resolution 177/2017 imposed a complete ban on paraquat starting in 2020 [75]. The preceding three years were a phase-out period to allow businesses to gradually stop paraquat use [3, 40].

China, the largest paraquat producer in the world, announced its intention to ban paraquat in 2012 due to its chronic toxicity and adverse health events, including suicides. In 2014, the government stopped registration and licensing of the liquid solutions of paraquat; all domestic sales and use of paraquat stopped in 2016. A paraquat gel product was registered in 2016 and withdrawn in 2020. The production of paraquat for export purposes is still allowed [76].

China's regulations banning hazardous organophosphorus insecticides and paraquat were associated with significant declines in total suicide deaths from 2006 to 2018. There was a decrease in suicide after the policy intervention in 2012 and a steeper decrease after the full ban of liquid formulations in 2016. The age-standardized suicide rate in China declined by 45.1%, from 12.70 in 2006 to 6.98 per 100.000 in 2018, while the age-standardized pesticide suicide rate decreased by 60.5%, from 6.50 to 2.56 per 100.000 [77].

Fiji’s first paraquat regulations came from a private party; in 2012, the Fiji Sugar Corporation banned the use of paraquat on sugarcane farms [78]. The government followed suit and banned paraquat beginning on 01 January 2020, citing negative effects on human health and the environment, including high numbers of suicides. From that date, the importation, sale, and use of paraquat in Fiji have been banned.

In India, one paraquat ion active ingredient, paraquat dimethyl sulfate, is banned at the central government level [79]. Several Indian states have introduced state-level limited regulations for remaining paraquat products within the powers given to them by the 1968 Insecticide Act. For example, Kerala banned all paraquat products in 2011, citing harm to human health [80, 81]; however, this regulation has recently been struck down by Kerala’s state’s Supreme Court. In 2023, Odisha temporarily banned paraquat, citing public safety reasons and the desire to prevent the adverse impact of the chemical on human health and animals [82]. The sale, stock, distribution, manufacturing, and use of the herbicide were prohibited in the state for a period of 60 days. The state government stated its intention to propose a permanent ban on paraquat to the central government.

In 2002, Malaysia’s Pesticide Control Division of the Agriculture Department ordered an end to registration and reregistration of paraquat products. A full ban on the use of paraquat was introduced in 2005. However, in 2006, after a campaign by the industry, the ban was reversed and replaced by regulations requiring dilution of paraquat products to reduce their toxicity [83]. Consequently, there was a five-fold increase in the number of paraquat poisonings and deaths, with the annual number of deaths from paraquat poisoning increasing from 34 in 2006 to 187 in 2015 [84]. The paraquat ban was reinstituted in 2020, with the use of pre-existing stocks in the plantation sector allowed until the end of 2020. Early studies of the impact of the ban showed that while paraquat was still the leading pesticide associated with mortality in 2015–2021, accounting for 66.7% of all fatal poisoning cases and 31.6% of all poisoning cases, the proportion of poisoning cases caused by paraquat decreased from 35.8 to 24%, and the overall case fatality decreased from 21.2 to 17.3% in the first year after the ban [83].

In Nigeria, a four-year phase-out plan for paraquat was announced in 2020 by the National Agency for Food and Drugs Administration and Control, with the importation to stop on December 31 st, 2022 [85]. The government agency was particularly concerned with the potential of paraquat to contaminate water sources, its persistence in the soil, and its high toxicity to humans. Paraquat is fully banned beginning on January 1, 2024, due to its detrimental effects on human health and the environment.

Since the 1990 s, South Korea has proceeded with increasingly restrictive paraquat regulations to address the high numbers of poisonings. In 1999, the government adopted the Paraquat Implementation Plan (PIP) and the Act on Paraquat Regulations, which labeled paraquat products"restricted use products"[23]. Although accidental ingestion decreased, intentional self-poisoning increased, and in 2011, the government canceled the re-registration of all paraquat products, banning the use of paraquat in 2012 [72]. A total of 11 paraquat liquid products were banned, after which all remaining products had to be taken back from sellers’ shops [32, 35].

Despite the changes in paraquat formulation, Sri Lanka has continued to experience high numbers of deaths from intentional poisoning with paraquat. A three-year phased ban on paraquat was implemented in 2009–2011 [30, 86]. Between 2011 and 2015, the age-standardized overall suicide rate decreased by 21%, from 18.3 to 14.3 per 100,000 people. The age-standardized rate of pesticide suicide decreased by 50% (from 8.5 to 4.2 per 100,000) [22].

In Taiwan, paraquat was listed as a highly hazardous pesticide (HHP) in 1983 due to the high number of acute poisonings [37, 38]. The Agro-Pesticides Management Act (2007) required that HHPs be stored in a locked cabinet in shops and that vendors keep records of who bought HHPs. However, these measures did not succeed in significantly reducing paraquat poisonings and deaths [37]. In 2017, the Council of Agriculture, Taiwan’s pesticide regulator, adopted a two-phase plan to ban products containing paraquat: first, a ban on the import and production of paraquat was introduced in 2018; second, a ban on the sale and use of the herbicide was announced beginning in 2019. This second phase of the ban was later postponed to February 2020 due to the high amounts of paraquat-containing pesticides left in stock [37, 38].

In Thailand**,** initial attempts to ban paraquat due to its toxicity and potential human health effects were met with opposition from powerful stakeholders [66, 87]. However, a paraquat ban became possible after a research group’s findings about harm to health and the environment were made public and following an extensive civil society and public discussion on the directions of pesticide policy in the country [66]. In 2019, paraquat was added to the Type 4 list of Thailand's Hazardous Substances Act, prohibiting the production, import, export, distribution, and possession of the herbicide. In October 2019, Thailand’s Hazardous Substance Committee voted in favor of banning paraquat; a paraquat ban entered into force in 2020 [88].

Vietnam designated paraquat for “restricted use” in 1999, but in 2001, all restrictions were withdrawn [68]. In 2017–2019, due to the renewed concerns of harm to health and the environment, the Ministry of Agriculture and Rural Development adopted regulations aimed at reducing and eliminating HHPs from use. In 2019, paraquat was removed from Vietnam’s List of Permissible Agrochemicals, effectively banning its use in the country [89].

### Regional stories

In territories where regional agencies and institutions include agricultural policies in their mandates, pesticide regulations exist at the regional level. In the European Union (EU), paraquat was banned in 2007 after the Court of First Instance of the European Communities annulled Directive 2003/112/EC amending Directive 91/414 to include paraquat as a plant protection substance [90]. The legal challenge was brought jointly by Sweden, Denmark, Austria, and Finland. In its judgment, the Court noted that before authorization of a substance for use, it must be established beyond a reasonable doubt that its use will be consistent with the protection of health. Based on the conditions of use, the Court ruled that there have been breaches of the principle of integration, the precautionary principle, and the principle requiring a high level of protection of the environment and human health [90]. Following this decision, members of the European Union that had not previously banned paraquat withdrew paraquat from its markets.^4^ Several European countries that are not part of the EU have also banned paraquat to harmonize their legislation with that of the EU (i.e., Bosnia and Herzegovina and Montenegro). The United Kingdom was part of the EU at the time of the 2007 decision, and so, paraquat was banned. Paraquat use has not been permitted since its exit from the EU.

In West Africa, the Permanent Interstate Committee for Drought Control in the Sahel (CILSS– an international organization uniting 13 countries^5^) established the Sahelian Committee for Pesticides (Comité Sahelién des Pesticides or CSP) in 1994 [91]. This regional body is responsible for implementing the Common Regulations on Pesticides in CILSS member states (1999). Only products approved by the CSP are authorized in its members'territory. In 2011, the CSP introduced regulations banning paraquat use in all its member states (Ministerial Decree from 5 August 2011) [48].In 2008, with the joining of other West African (ECOWAS) states (Ghana, Liberia, Nigeria, and Sierra Leone), the West African Regional Pesticide Registration Committee was launched. It is hoped that common pesticide regulations, including a paraquat ban, will be followed by new members, improving the situation across the whole region. However, it has been reported that countries in the region have difficulties removing paraquat from the market. Unregistered paraquat products are widely available in Burkina Faso and Mali, leading to poisonings and deaths [92–94].

The Cooperation Council for the Arab States of the Gulf^6^ adopted the Pesticide Law and its implementing Regulation in the Gulf Cooperation Council in 2015 [95]. This document harmonizes the legislation in the region and adopts a unified list of pesticides that are banned, which includes paraquat. Some neighbouring states (i.e., Lebanon) have also banned paraquat (Supplementary file). We found no account of successes or challenges in the implementation of this regulation.

### The international story

Difficulties in reaching international consensus on paraquat have kept it excluded from Annex III of the Rotterdam Convention on the Prior Informed Consent Procedure for Certain Hazardous Chemicals and Pesticides in International Trade (RC). Annex III contains a list of chemicals that have been banned or severely restricted for health or environmental reasons by two or more parties and that require prior informed consent (PIC) for trade. In 2011, the Chemical Review Committee (CRC) of the RC examined two formulations of paraquat and recommended their inclusion in Annex III of the Convention, concluding that the criteria for listing were met (for formulations EC and SL containing 200 g or more of paraquat ion/L). This occurred because the Burkina Faso government submitted a request to list it as a Severely Hazardous Pesticide Formulation (pesticides known to produce severe health or environmental effects observable within a short period after single or multiple exposure, under conditions of use). The Conference of the Parties discussed this proposal at multiple Conferences of the Parties to the Rotterdam Convention (decisions RC-3/3, RC-4/4, RC-6/8, RC-8/6, RC-8/7, and RC-9/5) but failed to reach a consensus. The listing of the chemical was repeatedly blocked by countries concerned with the lack of alternatives, their possible high costs, and alleged negative implications for trade. Paraquat-producing companies also insisted that paraquat is safe if it is used under conditions of use, ignoring the evidence of major harm due to easy access in farming communities [96].

In 2022, following an application from Malaysia and Mozambique, the CRC again reviewed a suggestion to include all products with paraquat active ingredient in Annex III and concluded that the active ingredient meets the criteria for listing banned or severely restricted chemicals under the Rotterdam Convention [97]. This latest decision applies to the active ingredient as a whole and will be discussed at one of the upcoming meetings of the Conference of Parties.

### Challenges in the adoption and implementation of paraquat regulations

A range of difficulties in the adoption and implementation of paraquat regulations was revealed by the systematic review. Some of these challenges were related to generating consensus among decision-makers on the need for regulation [23, 34, 50, 86]. Several papers have reported that paraquat regulations were reversed due to stakeholder pressure. One example was the 1991 paraquat ban in the Dominican Republic, after which agrochemical companies successfully argued that the herbicide posed no serious health effects and was necessary because of the high labor costs of manual weeding [73]. Consequently, paraquat's regulatory status was changed from"banned"to"restricted", and it is reportedly widely used throughout the country [73]. Before the recent bans in Malaysia, Thailand, and Taiwan, attempts to regulate the chemical had been challenged and reversed by the opposing parties.

Stakeholder concerns around paraquat bans are concentrated around economic and productivity losses if paraquat is banned, for example, not being able to carry the cost of other herbicides or hire help for manual weeding, the lack of other chemical methods of weeding, and the lack of knowledge of non-chemical alternatives (despite the presence of many alternatives) [25]. The reviewed case studies highlighted, however, that these concerns can be successfully addressed. The literature has shown that paraquat is quickly replaced by other herbicides or other methods of weed control (i.e., South Korea, Sri Lanka), proving that its ban has not had a significant impact on agricultural practices [36, 71]. Alternative methods of weed control without losses in agricultural yield or economic production are found [25, 98].

Some of the stakeholders’ hesitancy related to the paraquat ban was fuelled by the lack of data on exposure and poisoning [1, 27, 43, 99]. Surveillance of poisonings and knowledge of the harms associated with paraquat were vital for building consensus around pesticide regulations and for ensuring their implementation [50]. The more farmers are aware of the negative impacts of pesticide use, the less likely they are to oppose pesticide regulation and try to use banned pesticides [100]. Case studies from Taiwan and Thailand have indicated that improving data collection on pesticide poisoning and starting a public dialogue on the importance of preventing exposure and poisoning are pivotal in achieving policy change [37, 66]. In Thailand, as a high level of paraquat toxicity and harm to health has become known, public dialogue has resulted, contributing to stakeholder agreement with the proposed regulation [66]. In contrast, perceptions of pesticide use as a modern technique necessary for agricultural productivity and higher yields encourage pesticide use by farmers [101, 102].

The need to further strengthen the implementation of regulations was repeatedly highlighted in the reviewed literature [34, 51, 68, 69, 103]. Several papers noted that the harmonization of national and regional legislation in neighbouring and partnering countries could be an effective way of pooling limited resources to strengthen the implementation of pesticide policy.

The control of illegal smuggling of paraquat from neighbouring countries where paraquat is still legally sold or produced has been highlighted as an enforcement problem by several papers. If illegal trade in pesticides occurs within a country, regulatory enforcement authorities must understand the sources of the black market. Limited custom controls on exports and imports, limited training for border control agents and customs officials, multiple border crossings, and open borders were cited as challenges to enforcement [100]. It was reported that customs officers and frontline law enforcement officers were rarely trained to detect and recognize chemicals; they lack the resources to check products against their authorized specifications and may not be aware that a substance is illegal [100]. For example, while the sale of paraquat has been banned in French Guiana since 2007, this pesticide remains freely available in neighbouring countries, making it accessible in French Guiana [50] and responsible for many deaths from pesticide poisoning [104, 105]. Stricter implementation of restrictions on the sale and use of paraquat and strengthening of institutional capacity were highlighted as necessary to overcome these challenges [69, 104, 106, 107].

Recommendations in the reviewed literature included strengthening regular data collection on the health and environmental impact of pesticides [108]. The involvement of all stakeholders in decision-making based on evidence-based contextualized studies on the impact of pesticides and the deepening of public dialogue on the necessity of using pesticides and meaningful protective measures are recommended for achieving pesticide policy effectiveness [68, 103]. Since many agencies may be involved in the implementation of regulations, multi-stakeholder participation in the adoption and implementation of regulations is necessary, where Ministries of Agriculture, Ministries of Health and the Environment, customs and trade authorities share the mandate and responsibility and cooperate in achieving the overall goals of the regulations [109].

## Discussion

This systematic review showed that many strategies have been used to reduce the harmful health effects of paraquat. These strategies included lowering the concentration of the product, adding safening agents, and attempting to improve storage; restrictions on access to, sale, and use of the herbicide; and phase-outs and/or bans of paraquat [42]. Decreases in poisonings and deaths were mainly associated with paraquat phase-outs and bans, while restrictions—particularly in LMICs– were met with implementation difficulties. Targeted bans and phase-outs of paraquat resulted in significant reductions in accidental and intentional poisoning, often accompanied by a significant reduction in the overall suicide rate, without any apparent effect on agricultural yield.

Many of the reviewed experiences of the adoption and implementation of paraquat regulations point to the effectiveness of a phased approach [32]. During the phase-out period, manufacturing, import, export, distribution, and use are forbidden or limited, at the end of which the product is no longer authorized [37]. The phase-out period can be relatively long (e.g., a few years) or very short, leading to an almost immediate ban in the case of unacceptable risks. While a short phase-out period is very effective at reducing the existing risks, it may also lead to an accumulation of obsolete stocks and possible environmental contamination. A longer period may allow for the use of existing stocks in the supply chain but should be accompanied by enhanced risk mitigation measures (i.e., industry taking back pesticides from vendors and suppliers).

Regional regulations pull limited resources together for more effective pesticide management and allow for harmonized policies in countries with similar geographic, climatic, and economic circumstances. Furthermore, the effective implementation of regulations in one country will affect countries along its borders. A ban on paraquat in neighbouring countries and the elimination of the black market have been highlighted as important steps in tackling this challenge [51, 69, 104].

To overcome the challenges presented by regulating a popular herbicide, case studies suggested the importance of building consensus around stricter paraquat regulations among stakeholders and understanding the reasons behind the proposed bans. Pesticide users, vendors, advisors, and producers/importers need to be on board with the proposed regulations [108]. Engagement with public and private actors and awareness programs were important according to the experience of regulators who successfully introduced and implemented the regulations [31]. These activities form the support base for the decision and help create intersectoral cooperation during their adoption and implementation. Public discussions highlighting the reasons for regulations and enhancing stakeholders'knowledge of the harms associated with paraquat were important in providing legitimacy to the policy [99, 104].

When the public, including paraquat users, knows about the motivations for paraquat and other pesticide bans, they are more willing to implement them. Compliance is more likely if farmers become aware of damage to the environment and health [102, 110]. Knowledge of the alternatives and evidence for successful substitution with less toxic products serve as important factors in motivating users to stop paraquat use and should precede and accompany decision-making [81, 98].

While cost-effective alternative herbicides to paraquat, as well as a variety of non-chemical options, are available, depending on the crop and mode of use [25], many farmers are concerned about finding an effective alternative product. There is a concern that alternatives will be less effective or/and more expensive than paraquat at controlling weeds. Therefore, there is an important need to educate stakeholders on these issues, as farmers and their representative bodies need to be confident in the availability of alternatives [111–114].

Similarly to paraquat, another highly popular herbicide’s impact on health has recently generated intense attention and discussions. In contrast with paraquat, which is acutely toxic and has (disputed) chronic toxicity (association with Parkinson’s disease), glyphosate is not acutely toxic but is associated with chronic toxicity. Assessments of glyphosate’s harm to health differ depending on data sources and methodology used for classifications. In 2015, the International Agency for Research on Cancer (IARC) classified glyphosate as “probably carcinogenic to humans”, with reference to non-Hodgkin lymphoma, while other agencies (European Food Safety Authority and the US Environmental Protection Agency) came to different conclusions [115]. As a result of this debate, several countries have banned glyphosate using the precautionary principle. However, a ban on a herbicide as popular as glyphosate is perceived to make banning another popular herbicide– paraquat– more difficult. This does not need to be the case, as other alternative methods of weed control are available [25].

One of the barriers to adopting measures to reduce and eliminate the use of paraquat is its status as a WHO class II toxicity product, despite its high acute toxicity to humans [12]. To address this shortcoming, when available, WHO should use data on human toxicity, rather than rat or mice toxicity, when classifying pesticides by toxicity.

The current level of knowledge on acute human toxicity of paraquat and evidence of its health impact on humans should be sufficient to introduce bans at global, regional, and national levels. If in 2007, the EU court used the precautionary principle to ban paraquat from the EU markets, there currently is no need to use the precautionary principle in relation to paraquat’s acute toxicity. The precautionary principle states that if there is a risk of serious or irreversible harm to health or the environment, scientific uncertainty and lack of data should not be used as reasons to postpone preventive measures [116]. An abundance of evidence of paraquat’s high acute toxicity demands urgent preventative measures to preclude and eliminate its harm to health. Industry’s insistence on the lack of consensus on paraquat’s chronic and long-term effects should not be used as a reason to discount its acute toxicity and postpone preventative measures and its elimination from the market.

The review showed that the influence of the pesticide industry proved pivotal in decision-making on retaining and sometimes reintroducing paraquat at national markets. This influence can be further discussed within the framework of Commercial Determinants of Health (CDoH), which analyses strategies and approaches used by powerful private actors to promote the continued use of their products and foster choices that are detrimental to health [117, 118]. The CDoH framework is helpful in understanding the influence on health of tobacco [119], gambling [120], alcohol [121], unhealthy food [122] and pesticide industries. Insisting that paraquat use is safe, and claiming lack of viable alternatives is identified as common strategies used by the pesticide industry to avoid evidence-based policies [123]. One of the recommendations developed by the international community in relation to countering the harmful influence of private actors, and following the United Nations Guiding Principles on Business and Human Rights [124], is that if industry is allowed to participate in decision-making, such participation should be conducted transparently and responsibly, and the resultant policymaking should be consistent with public interest and respect for human rights, especially where the modes of engagement and the surrounding regulatory contexts impede adequate oversight [125–127].

Instead of continuing to insist on the safe use of paraquat, which is impossible in many LMIC settings [123], to fulfill their responsibility to protect health and the environment, paraquat producers need to consider the overwhelming evidence of paraquat’s high acute toxicity to humans and work on developing less toxic alternatives to this herbicide.

Considering that many societies pay little attention to the impact of pesticides on the health and wellbeing of the affected communities, the impact of paraquat use is not known where researchers do not address this issue specifically, and where targeted funding is not available. A study of paraquat regulations in the regions where there are gaps in knowledge on its use and impact on health is recommended to address this shortcoming.

The regulatory responses to paraquat problems have been diverse but follow a pattern where less strict methods of addressing the harm are tried first (formulation changes and restrictions), prior to using the most effective method– its elimination from the market. Reported difficulties in enforcing restrictions and the resultant continuing harm to health show that, in countries with high numbers of poisonings and deaths, it may be useful to learn from similar experiences in other jurisdictions and save lives and health by proactively banning this toxic pesticide from use.

### Strengths of our review

The are several strengths to this review.

We have not only searched peer-reviewed literature on paraquat regulations, bans, and restrictions, we included in our review a broader subject of pesticide regulation, bans, and restrictions to account for the paucity of literature on paraquat regulations. Including the papers that concentrated on a broader subject of pesticide regulations allowed us to review a wider range of publications to find information about paraquat regulations.

Recognising the scarcity of literature on paraquat regulations, dominated by the discussions of bans in several widely researched countries, we included grey literature and searched regulatory authorities reports, international agencies documents, and circulars. We included primary sources of legislation, regulations, and rules on paraquat. We reviewed regulatory websites of major pesticide-using and producing countries accessible to English- speaking researchers. We followed up information on paraquat restrictions and bans available on the website of the RC and contacted select regulators from the contacts available on the FAO website. This provided a major strength to the study as it gave us access to not previously published information.

### Limitations

Due to resource limitations, we did not include papers and grey literature that were not in English. This may have limited our results as some relevant studies may not have been identified. We also did not review national regulators’ websites or documents that were not accessible to English speakers.

Some countries that do not authorise paraquat were likely missed from the review due to an absence of published reports or analysis of paraquat regulations. Furthermore, while we collected data on bans and phase-outs of paraquat and believe that our dataset was the most exhaustive at the time of the study, we did not collect exhaustive data on restrictions on paraquat. It appears that most countries that still use this herbicide do so with restrictions on its use. However, our list of restrictions is not exhaustive, providing only examples of such measures.

Several Private Standards have limited or banned the use of paraquat in their commodities (Rainforest Alliance and Fairtrade in coffee and pineapples [25]). These private voluntary standards are not covered by this review.

## Conclusion

The systematic review showed that many countries have realized the public health and environmental harms associated with paraquat and have chosen to strictly regulate or ban it. Bans, if adopted following stakeholder agreement and support, show a remarkable decrease in paraquat mortality and, sometimes, in suicide mortality in general. Recommendations for action needed to decrease and eventually eliminate paraquat poisonings and deaths included toxicology and environmental surveillance of paraquat poisonings to provide reliable data, regulatory coordination between countries to save valuable enforcement resources, and rigorous implementation of regulations, including training for enforcement officers and regular monitoring of domestic markets to identify violations. Most importantly, national and international decision-makers and policy-makers need to take seriously the health consequences of paraquat exposure, and the poisonings and deaths associated with its use. Paraquat needs to be eliminated from use, particularly in countries where its use causes the most harm. It is time that governments put people’s health and lives first above the misplaced fears of productivity loss and put a firm stop to the use of the deadly herbicide.

## Supplementary Information

Supplementary Material 1.

Supplementary Material 2.

## Data Availability

Data is provided within the manuscript or supplementary information files.

## Footnotes

1 Syngenta manufactures paraquat at its factory in Huddersfield, West Yorkshire, although its use has been banned in the country since 2007. Syngenta ships paraquat to the US, Colombia, Mexico, Ecuador, Guatemala, and India. Crispin Dowler, Revealed: UK shipped more than 10,000 tons of banned pesticides overseas in 2020. UNEARTHED. https://unearthed.greenpeace.org/2022/02/22/bees-syngenta-paraquat-uk-exports/.

2 Paraquat has a dedicated information center linked to Syngenta https://www.paraquat.com/en/

3 It is reported that paraquat ban was instituted in Finland in 1985. Andrew Watterson “Pesticide Users’ Health and Safety Handbook: an International Guide”, Gower Technical (1988).

4 Members of the EU in 2007 were: Germany, France, Italy, the Netherlands, Belgium, Luxembourg, Denmark, Ireland, United Kingdom, Greece, Spain, Portugal, Austria, Finland, Sweden, Chechia, Cyprus, Estonia, Latvia, Lithuania, Hungary, Malta, Poland, Slovenia, Slovakia, Bulgaria, Romania. Croatia became the 28th EU member in 2013. In 2020 UK exited the EU. https://european-union.europa.eu/principles-countries-history/history-eu_en

5 Members of the CILSS are Benin, Burkina Faso, Cape Verde, Chad, Gambia, Guinea, Guinea-Bissau, Ivory Coast, Mali, Mauritania, Niger, Senegal, and Togo. Other ECOWAS states are Ghana, Liberia, Nigeria, and Sierra Leone.

6 Members of the Gulf Cooperation Council are the United Arab Emirates, Kingdom of Bahrain, Kingdom of Saudi Arabia, Sultanate of Oman, State of Qatar, and State of Kuwait (https://www.gcc-sg.org/en-us/AboutGCC/MemberStates/Pages/Home.aspx).
